# The Effect of Adipose-Derived Stem Cells on Full-Thickness Skin Grafts

**DOI:** 10.1155/2016/1464725

**Published:** 2016-06-20

**Authors:** Juan Wang, Haojie Hao, Hong Huang, Deyun Chen, Yan Han, Weidong Han

**Affiliations:** ^1^Department of Plastic and Reconstructive Surgery, Chinese PLA General Hospital, Beijing 100853, China; ^2^Medicine School of Chinese PLA, Beijing 100853, China; ^3^Institute of Basic Medicine Science, College of Life Science, Chinese PLA General Hospital, Beijing 100853, China; ^4^School of Medicine, Nankai University, Tianjin 300071, China

## Abstract

*Background.* The purpose of this study was to evaluate the effects of ASCs on full-thickness skin grafts. Specifically, we investigated the anti-inflammatory effects of ASCs that are mediated via regulation of the phenotypes of activated macrophages.* Methods.* ASCs were isolated, cultured, and injected under full-thickness skin grafts in 15 rats (ASC group). An additional 15 rats served as controls (PBS group). Skin graft survival assessment and vascularization detection were assessed with H&E staining and laser Doppler blood flowmetry (LDF). The effects of ASCs on angiogenesis, anti-inflammation, collagen accumulation-promoting, and antiscarring were assessed.* Results.* We found that the skin graft survival rate was significantly increased in the ASC group. The neovascularization, collagen deposition, collagen type I to type III ratio, and levels of VEGF and TGF-*β*3 in the ASC group were markedly higher than those in the PBS group at day 14. Additionally, in the ASC group, the levels of iNOS, IL-1*β*, and TNF-*α* were remarkably decreased, whereas the levels of IL-10 and Arg-1 were substantially increased.* Conclusions.* Our results confirm that ASCs transplantation can effectively improve full-thickness skin graft survival. Additionally, the anti-inflammatory role of ASCs may indirectly contribute to skin graft survival via its effect on macrophage polarization.

## 1. Introduction

Skin grafts are the preferred approach for the treatment for large skin defects in plastic and reconstructive surgery. Full-thickness skin grafts consist of epidermis, dermis, and subcutaneous tissue with epidermal appendages and have the advantages of resisting contracture, reconstructing defect volumes, and contributing to improved texture and coloration matching compared with split-thickness skin grafts. However, the increased thickness is less conducive to plasmatic imbibition, which can directly influence revascularization and graft survival during the first 24 to 48 hours [1] and consequently result in partial or complete necrosis and a low survival rate of full-thickness skin grafts. Therefore, novel and effective therapies for promoting full-thickness skin graft survival are needed.

During the past decade, adipose-derived stem cells (ASCs), which are one type of the mesenchymal stem cells (MSCs), have undoubtedly provided a new therapeutic method in terms of angiogenesis and tissue regeneration due to the self-renewal, multidirectional differentiation ability, easy collection, and weak immunogenicity characteristics of ASCs [2]. Previous studies indicate that the angiogenesis mechanisms of ASCs that function in tissue repair and wound healing cannot be solely ascribed to the differentiation of ASCs into endothelial lineages [3, 4] but also involve paracrine effects mediated by the secretion of numerous cytokines and growth factors, like VEGF, HGF, bFGF, and others [5, 6]. Based on above mechanisms, ASCs are widely recognized to play key roles in the promotion of wound healing and skin flap survival [7, 8]. Moreover, the role of ASCs in full-thickness skin grafts in rats has been explored by Zografou et al. [9]. However, the differentiation concentration of ASCs may be limited due to low-efficiency differentiation [10]. Furthermore, there are also long-standing controversies regarding whether ASCs directly differentiate into endothelial cells [11, 12]. Thus, the paracrine signaling mechanism seems to be more important in angiogenesis than the direct differentiation mechanism [13]. Additionally, studies have also demonstrated that MSCs can promote the proliferation, migration, and collagen secretion of fibroblasts through a paracrine mechanism independently of the promotion of angiogenesis [14].

Moreover, in recent years, numerous lines of evidence have highlighted the modulatory effects ASCs exert on macrophages, which involve the polarization of macrophage from the M1 phenotype (proinflammatory) to the M2 phenotype (anti-inflammatory). This polarization is an important regulator during the processes of angiogenesis and tissue regeneration due to the release of angiogenic cytokines and the maintenance of hemostasis through anti-inflammatory cytokines such as IL-10 [15–17]. Coculturing with MSCs induces the conversion of monocytes or macrophages to the M2 phenotype via increased expression of IL-10 and reduced expression of TNF-*α* and IL-1*β* [18]. Donizetti-Oliveira et al. reported that ASCs prevent renal disease primarily via the anti-inflammatory effects [19]. Hao et al. demonstrated that ASCs create a suitable microenvironment for tissue regeneration by polarizing IL-10-releasing M2 macrophages in hindlimb ischemia [20]. Dong et al. reported that the adipose stromal vascular fraction (SVF) can improve the transplanted adipose tissue survival by converting macrophages into angiogenic and anti-inflammatory M2 macrophages [21]. However, to date, there are no reports about the anti-inflammatory mechanism of ASCs in full-thickness skin graft models.

Therefore, the precise mechanism of the action of ASCs on full-thickness skin grafts is still not fully understood, particularly in terms of the potential anti-inflammatory mechanism, which has not been reported. The aim of this study was to verify the effect of ASCs on full-thickness skin grafts. Specifically, we emphasized the anti-inflammatory effect of ASCs that is mediated by an increase in M2 macrophages. Our research will provide a comprehensive understanding of the function of ASCs in full-thickness skin grafts.

## 2. Methods

All procedures of the present study were performed in accordance with the Chinese PLA General Hospital Animal Care and Use Committee's guidelines for the principles of animal care.

### 2.1. Isolation and Culture of ASCs

ASCs were harvested from the inguinal fat pads of Sprague-Dawley (SD) rats weighing approximately 80 g each. In brief, according to the protocol described by Zhang et al. [22], the harvested rat adipose tissue was extensively washed with phosphate-buffered saline (PBS) and then finely minced and digested for 45–50 min at 37°C with type I collagenase (Sigma, St. Louis, USA). Next, the collagenase was neutralized with low-glucose Dulbecco's modified Eagle's medium (DMEM; Gibco Life Technologies, USA) supplemented with 10% fetal bovine serum (FBS; HyClone Laboratories, USA) and 100 U/mL penicillin/streptomycin (Gibco Life Technologies, USA). The cell suspension was then followed by filtration through a 200 *μ*m nylon mesh and centrifugation at 400 g for 5 min. Cells were resuspended and plated in 25 cm^2^ cell culture flasks and maintained at 37°C in 5% CO_2_ and 95% humidity. Cells in the 3rd passage were used in the subsequent experiments.

### 2.2. Identification of ASCs

The morphologies of the ASCs were observed under a phase-contrast microscope (Olympus IX71). According to a previously described protocol [23, 24], the immunophenotypes of the ASCs were determined by flow cytometry-based evaluations of CD11a, CD34, CD73, CD90, CD105, and HLA-DR, and the multipotencies of the cells were confirmed by their adipogenic, osteogenic, and chondrogenic differentiations. The ASCs were cultured in adipogenic, osteogenic, and chondrogenic differentiation medium (Cyagen Biosciences Inc., USA), respectively, and the medium was changed every 3 days. After 2 to 3 weeks, the ASCs that differentiated into adipocytes, osteocytes, and chondrocytes were confirmed by staining with oil-red O, alkaline phosphatase, and Alcian Blue, respectively.

### 2.3. Full-Thickness Skin Graft Model and Cell Transplantation

Thirty male SD rats weighing between 250 and 350 g were randomly divided into 2 groups (*n* = 15), that is, a phosphate-buffered saline (PBS) group and an adipose tissue-derived stem cell (ASC) group, according to the local treatment used. Each group was equally divided into five subgroups of three rats each according to the time of observation at 1, 2, 3, 7, or 14 days after the operation. After anesthesia with 3% pentobarbital sodium (1 mL/kg intraperitoneal), a 3 × 3 cm^2^ full-thickness cutaneous tissue including panniculus carnosus was carefully removed in the midline of the back. The graft was then reattached to the underlying muscle fascia layer with 5-0 silk braided nonabsorbable suture (MERDILK). The sutures were intentionally left long. A bolster of cotton or dressing impregnated with medicinal alcohol was placed on the graft, which was sutured face to face. The restraining bandage (URGO Strapping) was then used to cover the surface of the bolster, which precisely adapted the skin graft onto the recipient bed to optimize the conditions for the revascularization process.

1 × 10^6^ ASCs resuspended in 0.5 mL of PBS were prepared. In the ASC group, 0.5 mL ASCs suspension was equally administered at 10 sites of the fascial layer of the recipient bed with a 1 mL syringe (26-gauge needle) prior to skin graft reattachment, while the control group received 0.5 mL of PBS in the same method. Subsequently, in order to track ASCs, the translated ASCs were dyed with CM-Dil (Invitrogen, Carlsbad, USA) at day 14 observation group. According to the manufacturer's recommendations, 5 *μ*L CM-Dil was added to 1 mL cell suspension with 1 × 10^6^ ASCs.

### 2.4. Skin Graft Survival Assessment

At days 1, 2, 3, 7, and 14 after the operation, the survival condition of the graft was grossly determined based on appearance, color, and texture. Next, the surviving and vascularized areas were detected and quantified with laser Doppler blood flowmetry (LDF; Perimed AB, Sweden) according to the instructions provided by the company. The blood perfusion unit (BPU) value was measured. The results were determined with the relevant Pimsoft software.

### 2.5. Specimen Collection and Detection

At days 1, 2, 3, 7, and 14 after the skin graft operation, the complete 3 × 3 cm^2^ full-thickness skin biopsy including the panniculus carnosus with a 0.5 cm margin was harvested. Next, each skin specimen was equally divided into two parts. One part was stored in liquid nitrogen for RNA extraction, and the other part was fixed with 4% paraformaldehyde for histological analysis.

### 2.6. Histologic Examination

Paraffin-embedded tissues from the skin grafts were sectioned at thickness of five micrometers and then mounted on adhesive glass slides. Hematoxylin and eosin (H&E) staining was performed according to standard procedures. Moreover, deparaffinized sections of day 14 were subjected to Masson's trichrome and picrosirius red staining to observe the collagen frameworks.

For immunohistochemistry analysis, deparaffinized sections of day 14 were incubated with VEGF polyclonal antibody (Abcam) and TGF-*β*3 (Abcam), respectively, at 4°C overnight and then followed by biotinylated goat anti-rabbit IgG antibody (Invitrogen) with PBS for 1 h. At last, visualization was performed with DAB (Sigma). Quantitative evaluations of TGF-*β*3 and collagen I and collagen III were assessed using Image Pro Plus 6.0 software (USA) according to mean density (IOD/area).

### 2.7. Immunofluorescence

After fixation with paraformaldehyde and dehydration with sucrose, tissues of days 1 and 2 were embedded in OCT. 5 *μ*m-thick section was incubated with primary antibodies against the M2 marker Arg-1 (Abcam) overnight at 4°C and then with the Alexa Fluor-conjugated IgG secondary antibodies (Santa) for 1 h at room temperature and finally followed by DAPI dye (Sigma) for 5 min. The cells were examined with a confocal imaging system (Olympus FV1200).

Moreover, the detection of the transplanted CM-Dil-labeled ASCs at day 14 was performed through immunofluorescence, and the tissues from day 14 were incubated with antibody vWF (Abcam) using the same method described above.

### 2.8. Real Time PT-PCR Analysis

Total mRNA was extracted from homogenized excised tissues using TRIzol reagent (Takara). Reverse transcription into cDNA was done using a cDNA synthesis kit (Takara) according to the manufacturer's instructions. Next, the quantitative real time PCR was performed with the SYBR Green Master mix (Toyobo) on an ABI Prism 7500 Sequence Detection System (Applied Biosystems). The relative fold changes in expression were calculated following normalization to *β*-actin [25]. The primer sequences are presented in Table 1.

### 2.9. Western Blotting

Proteins were isolated from homogenized excised tissues of day 1 and day 2 as previously described [25]. The total protein (30 *μ*g) of each sample was subjected to SDS-PAGE and immunoblotting with the antibodies against Arg-1 (Abcam) and *β*-actin (Abcam). The secondary antibodies were goat anti-mouse and rabbit anti-goat IgG (HRP).

### 2.10. Statistical Analysis

All data were expressed as the means ± standard deviations and were analyzed using SPSS 17.0 (USA). Independent Student's *t*-tests or one-way analyses of variance were used for comparison between 2 groups. In general, *p* < 0.05 was considered to be statistically significant.

## 3. Results

### 3.1. Characterization of the ASCs

**Figure 1:**
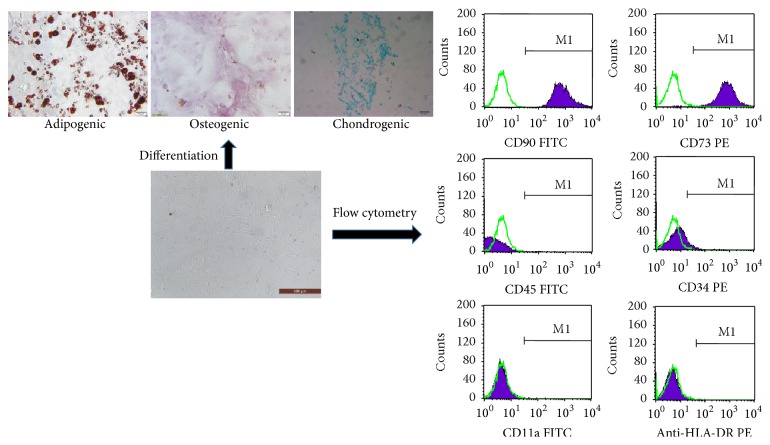
Characterization of ASCs. First, ASCs can be found plastic-adherent when maintained in standard culture conditions. Second, ASCs express CD73 and CD90 and lack expression of CD34, CD45, CD11a, and HLA-DR surface molecules. Third, ASCs differentiate to osteoblasts, adipocytes, and chondroblasts in vitro.

The cells that were isolated from the inguinal fat pads of the SD rats were found to be plastic-adherent and exhibited a spindle-shaped morphology when observed under a light microscope. Flow cytometry analysis revealed that the cultured cells were positive for the CD73 and CD90 and negative for the CD11a, CD34, CD45, and HLA-DR surface molecules. Additionally, the specific staining revealed that the ASCs were capable of differentiating into osteoblasts, adipocytes, and chondrocytes (Figure 1).

### 3.2. Skin Graft Survival Assessment and Vascularization Detection

**Figure 2:**
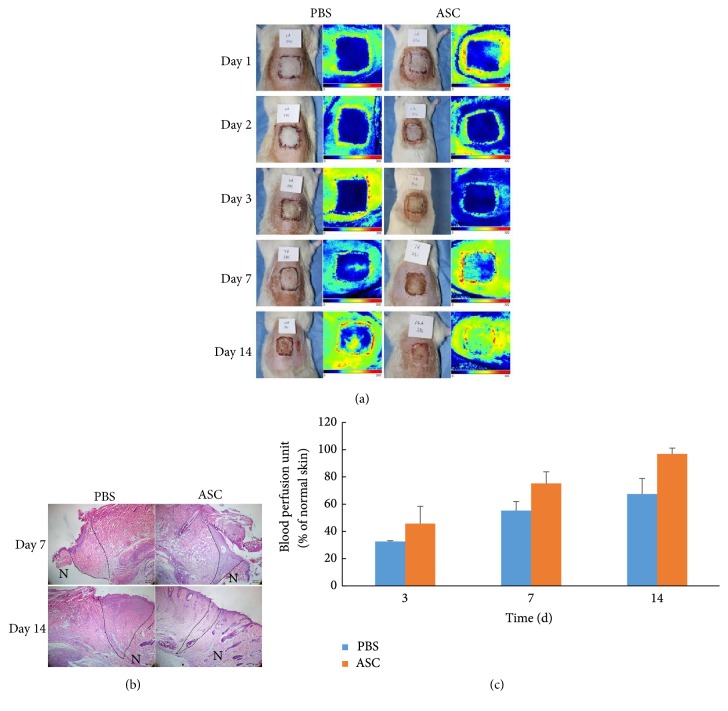
Observation of blood flow perfusion. (a) Perfusion images of skin grafts at days 1, 2, 3, 7, and 14 postoperatively. The color scale of all images was analyzed by setting the lowest perfusion value to 0 and the highest perfusion value to 300. Images showed blood perfusion images in PBS group and ASCs group, respectively. (b) H&E staining at day 7 and day 14 showed the survival condition of ASC group was much better in both the wound margin and the skin grafts. Scale bar 200 *μ*m. (The dashed area showed the wound margin. N presented normal skin, and the other side presented the skin graft.) (c) Blood flow perfusion in ASC group was higher than that in PBS group at days 3, 7, and 14. The blood perfusion was expressed as a ratio of the normal skin in the same area.

To determine whether the ASCs could improve the full-thickness skin graft survival, 3rd-passage ASCs were injected into the fascial layer of the recipient bed. On days 1, 2, 3, 7, and 14 after the operation, the surviving conditions of the grafts were difficult to definitely determine with the naked eye based on appearance, color, and texture, while H&E staining reviewed the difference survival condition of the skin graft between ASCs and PBS group at days 7 and 14 (Figure 2(b)). Survival condition of ASC group was much better than PBS group in both the wound margin and the skin grafts. Laser Doppler perfusion imaging was performed to detect the blood perfusion of the skin grafts (Figure 2(a)). The results were expressed as a ratio of the normal skin in the same area. The results revealed a time-dependent increase in blood perfusion. The average perfusion ratio was higher in the ASC group than in the PBS group at days 3, 7, and 14 (Figure 2(c)).

### 3.3. Anti-Inflammatory Effect of the ASCs in the Full-Thickness Skin Grafts

**Figure 3:**
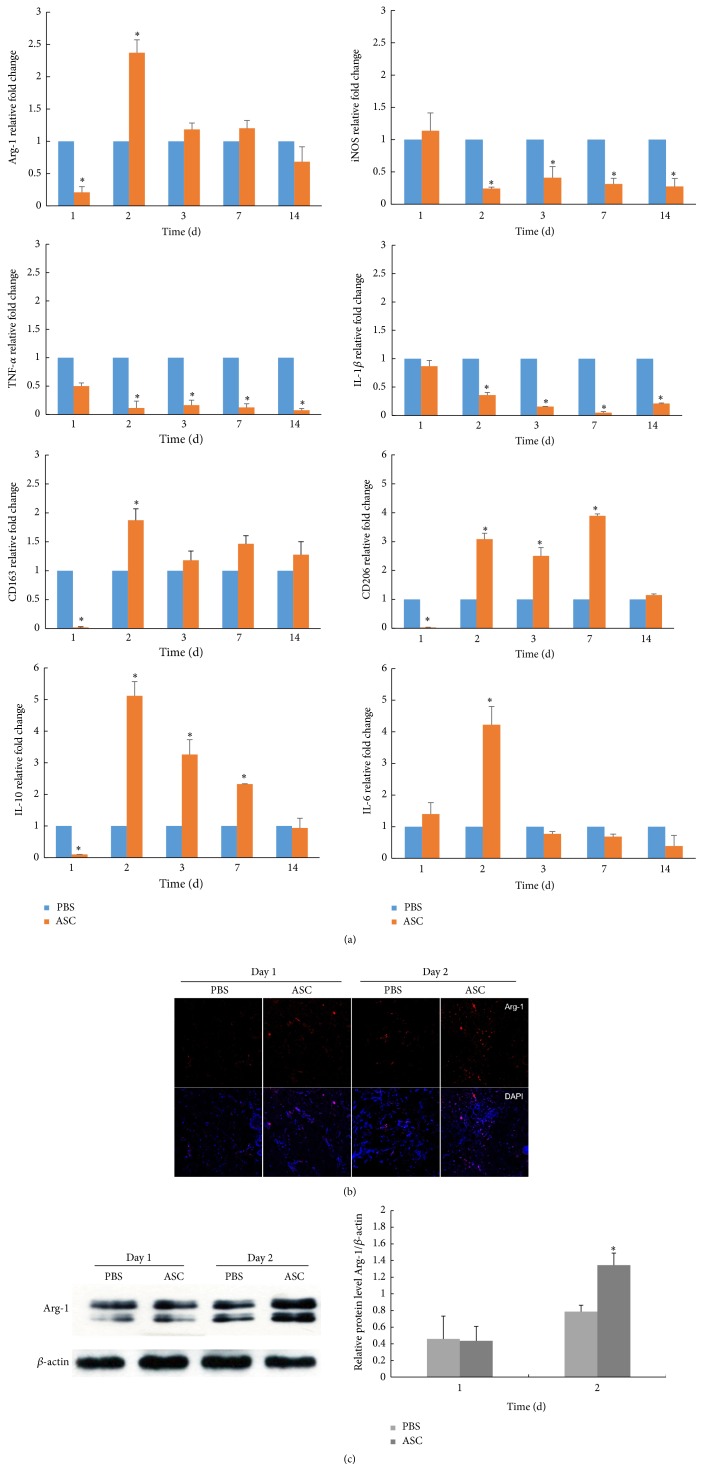
Anti-inflammatory effect of ASCs by converting macrophage to M2 polarization. (a) Proinflammatory cytokines (IL-1, IL-6, and TNF-*α*), anti-inflammatory cytokines (IL-10), and M1, M2 macrophages surface marker iNOS and Arg-1, CD206, and CD163 expression in skin grafts for various times. mRNA expression of interested cytokines is normalized to *β*-actin and given as relative change. Data are presented as the mean ± SD of three separate experiments. ^*∗*^Compared with PBS, *p* < 0.05. (b) Immunofluorescence analysis of M2 phenotype on days 1 and 2. The densities and distributions of M2 macrophages were significantly increased in the ASC group especially on day 2. Scale bar 100 *μ*m. (c) The protein level of Arg-1 at days 1 and 2 by western blotting.

To investigate the anti-inflammatory effects of the ASCs in the full-thickness skin grafts, we assessed the levels of inflammatory and inflammation-related cytokine expression on days 1, 2, 3, 7, and 14 by PT-PCR. The results revealed that the ASC group produced dramatically lower levels of proinflammatory cytokines (IL-1*β* and TNF-*α*) and the M1 macrophage surface marker iNOS and a higher level of the anti-inflammatory cytokine IL-10 and the M2 macrophage surface marker Arg-1, CD163, and CD206. However, the IL-6 level was significantly higher on day 2 in ASC group than in the PBS group (*p* < 0.05; Figure 3(a)). Furthermore, immunofluorescence for Arg-1 (M2 marker) on days 1 and 2 revealed that the densities and distributions of M2 macrophages were significantly increased in the ASC group especially on day 2 (Figure 3(b)). Moreover, the result was confirmed in protein level by western blotting (Figure 3(c)). Taken together, these data indicated that the ASCs acted as homeostatic regulators of inflammation and facilitated the expression of the anti-inflammatory phenotype (M2).

### 3.4. Effects of ASCs on Angiogenesis

**Figure 4:**
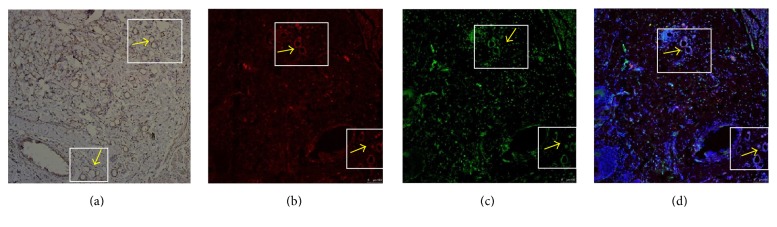
Detection of transplanted CM-DiI-labeled ASCs at day 14. (a) Immunohistochemistry with VEGF. Brown color indicates VEGF positive staining. (b) CM-DiI-positive ASCs arranged in a vessel-like structure (yellow arrows). (c) Immunofluorescence with vWF. (d) Image in (b) and (c) merged with nuclear stain by DAPI revealed involvement of CM-DiI-positive ASCs in skin graft neovascularization. Scale bar 100 *μ*m.

To determine the possible effect of the ASCs on angiogenesis in the skin grafts, immunohistochemical staining for VEGF was performed on day 14. The results revealed that the VEGF levels in the ASC group were significantly greater than those in the PBS group in both the skin and the cutaneous wound. Furthermore, vWF immunofluorescence was examined, and we found that several CM-Dil-labeled ASCs were arranged in a vessel-like structure that stained positively for vWF (Figure 4). These findings indicated that the ASCs resulted in an increase in angiogenesis in the full-thickness skin grafts.

### 3.5. Collagen Accumulation-Promoting and Antiscarring Effects of ASCs

**Figure 5:**
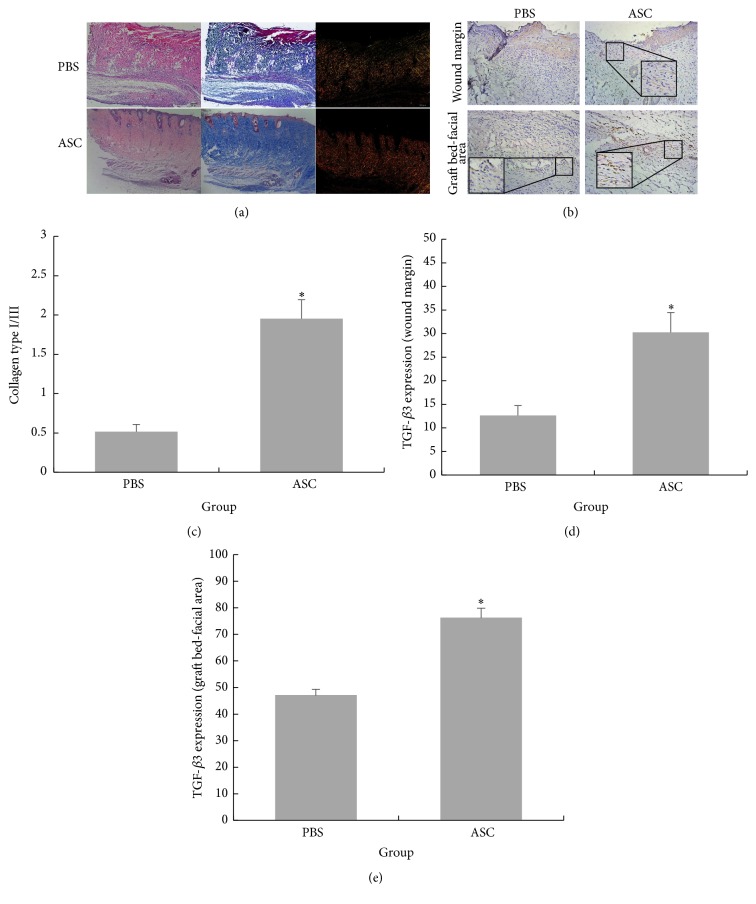
(a) The H&E staining and Masson's trichrome staining showed that more compact structure and more collagen deposition were found in ASC treated group compared with PBS treated group at day 14. This finding was also verified by exploring the distribution of collagen I and collagen III through picrosirius red staining. The ratio of collagen I and collagen III was much higher in ASCs treated group than that of PBS treated group. Scale bar 200 *μ*m. (b) Immunohistochemistry with TGF-*β*3 in both epidermis-dermis of the skin graft wound margin and graft bed-facial area. The TGF-*β*3 level was significantly higher than those in PBS group. Scale bar 50 *μ*m. (c) Quantitative evaluation of ratio of collagen types I and III. (d and e) Quantitative evaluation of TGF-*β*3 expression of the skin graft wound margin and graft bed-facial area, respectively. Data are presented as the mean ± SD of three separate experiments. ^*∗*^Compared with PBS, *p* < 0.05.

Furthermore, the effects of the ASCs on collagen deposition were estimated. Masson's trichrome staining on day 14 revealed an increased fibroblast density and increased collagen deposition in the ASC group, while irregular packing of collagen fibers was observed in the control group (Figure 5(a)). The ASCs promoted the deposition of collagen in the skin grafts. This finding was also verified by an exploration of the distributions of type I and III collagen through using picrosirius red staining. Compared with the PBS group, the ratio of type I to type III collagen in the ASC group was remarkably upregulated (Figure 5(c)). Obviously, the ASCs modified the accumulation of type I and III collagen on day 14 in the full-thickness skin grafts. Finally, we also detected TGF-*β*3 expression. The immunohistochemistry results revealed positive TGF-*β*3 staining in the epidermal keratinocytes, interfollicular cells, fibroblasts, and endothelial cells in both the ASC and PBS groups. Furthermore, the TGF-*β*3 levels in the ASC group were significantly higher than those in the PBS group in both the skin graft wound margin and the graft bed-fascia area (Figures 5(b), 5(d), and 5(e)).

## 4. Discussion

Compared with split-thickness grafts, the color, texture, and thickness of full-thickness skin grafts are more close to normal skin, and most importantly, full-thickness skin grafts undergo less contraction while healing. However, full-thickness skin grafts are limited to relatively small, uncontaminated, well-vascularized wounds and thus are not as widely applicable as split-thickness grafts in plastic and reconstructive surgeries. Thus, attempts to improve full-thickness skin graft survival are of undoubtable prospective clinical value. Full-thickness skin grafts undergo a well-known wound healing process comprising inflammation, proliferation, and maturation [26]. In this study, we found that ASC treated full-thickness skin grafts exhibited greater blood perfusion presumably because the ASCs decreased the inflammatory response, promoted angiogenesis, and modified the accumulations of the different collagen types at different time points.

The inflammatory phase peaks between 1 and 3 d after the injury and can last for 4–6 d in normally healing wounds. High levels of inflammation are well known to inhibit healing and induce scar formation. Generally, except promoting the removal of apoptotic and necrotic cells, macrophages also coordinate tissue repair processes, such as angiogenesis and scar formation, through the production of many cytokines and factors that stimulate angiogenesis, collagen synthesis, and fibrosis [27]. M2 macrophages have been proved to play important roles in wound healing and angiogenesis [28, 29]. In our study, both the RT-PCR and immunofluorescence results suggested that the ASCs appeared to improve the full-thickness skin grafts by improving M2 macrophage expression and regulating local inflammation. However, we noted that the upregulated genes included the proinflammatory factor IL-6. This finding is similar to those of several reports that have demonstrated that MSCs constitutively produce IL-6 and polarize monocytes (M0) toward anti-inflammatory M2 macrophages; in contrast, in the absence of IL-6, MSCs induce the polarization of M0s toward proinflammatory M1 macrophages [30, 31].

Revascularization is critical for long-term skin graft survival. Consistent with the results of Zografou et al. [9, 32], we found that ASCs can form vessel-like structures, which confirmed the differentiation effects. However, only a small number was found. Therefore, we emphasize the paracrine effects of ASCs in angiogenesis via the secretion of proangiogenic cytokines and factors, such as VEGF.

Currently, increasing numbers of studies are supporting the role of ASCs in anesthetic surgery, which include antiscarring and collagen deposition-promoting functions [33, 34]. Moreover, as we stated previously, the role of macrophage phenotype in fibrosis has been emphasized in several studies. Therefore, we examined the collagen deposition and expression of antiscar factor TGF-*β*3 following the treatment of full-thickness skin grafts with ASCs. Type I collagen is the predominant collagen and is responsible for the greater tensile properties of normal skin, whereas type III collagen is mainly found in early wound healing stages and is reabsorbed in the later stages [16]. The proper ratio of type I to type III collagen is essential during wound healing and plays vital roles in the prevention of scarring [35]. This ratio decreases from 4 : 1 to 2 : 1 due to an early increase in the deposition of type III collagen during wound healing [36]. In accordance with the above findings, the type I/III collagen ratio in the ASCs group at day 14 was much higher than that of the PBS group in our skin grafting model. Finally, in terms of antiscarring effects, the antiscarring factor TGF-*β*3, which is expressed at higher levels in fetal fibroblasts than adult fibroblasts [37], was upregulated in the ASC group at day 14 in both the skin graft wound margin and the graft bed-fascia area.

## 5. Conclusion

This report demonstrates that autologous ASC transplantation can effectively improve full-thickness skin graft survival by promoting angiogenesis and the accumulation of modified collagen types. Additionally, ASCs increased the proportion of anti-inflammatory M2 macrophages, which may have contributed to angiogenesis and full-thickness skin graft survival through indirect mechanisms. However, further experiments are needed to verify the anti-inflammatory effects of ASCs in terms of the role of polarized macrophages. Nevertheless, we believe that the present study provides helpful insight into the role of ASCs in full-thickness skin grafts and thus provides a theoretical foundation for further clinical applications of ASCs.

## Figures and Tables

**Table 1:** The primer sequence for real time PCR.

Gene	Forward	Reverse
IL-1*β*	5′-GAGAGGGAAATCGTGCGTGAC-3′	5′-ATCATCCCACGAGTCACAGAGG-3′
IL-6	5′-CCGGAGAGGAGACTTCACAG-3′	5′-TGACAGTGCATCATCGCTGTTC-3′
TNF-*α*	5′-TCCGCAGATACCTGGAACTC-3′	5′-CTCAGATCCTCCCCATTCAA-3′
IL-10	5′-ATGGCCCAGAAATCAAGGAGC-3′	5′-GAAGATGTCAAACTCATTCATGGCC-3′
iNOS	5′-CCAACCTGCAGGTCTTCGATG-3′	5′-GTCGATGCACAACTGGGTGAAC-3′
Arg-1	5′-CCAAGCCAAAGCCCATAGAG-3′	5′-TCCTCGAGGCTGTCCCTTAG-3′
CD206	5′-ACTGCGTGGTGATGAAAGG-3′	5′-TAACCCAGTGGTTGCTCACA-3′
CD163	5′-ACCCTTGAAAGTCTCCCATATCT-3′	5′-ATGACAGCCAGCTAAATGGACA-3′
*β*-actin	5′-GAGAGGGAAATCGTGCGTGAC-3′	5′-CATCTGCTGGAAGGTGGACA-3′
